# Tobacco Use Among Iraq- and Afghanistan-Era Veterans: A Qualitative Study of Barriers, Facilitators, and Treatment Preferences

**DOI:** 10.5888/pcd9.110131

**Published:** 2012-02-16

**Authors:** Jennifer M. Gierisch, Kristy Straits-Tröster, Patrick S. Calhoun, Jean C. Beckham, Shawn Acheson, Kim Hamlett-Berry

**Affiliations:** Center for Health Services Research in Primary Care, Durham Veterans Affairs (VA) Medical Center (152). Dr Gierisch is also affiliated with Duke University, Durham, North Carolina.; Mid-Atlantic Mental Illness Research, Education and Clinical Center, Durham VA Medical Center and Duke University, Durham, North Carolina; Mid-Atlantic Mental Illness Research, Education and Clinical Center, Durham VA Medical Center and Duke University, Durham, North Carolina; Mid-Atlantic Mental Illness Research, Education and Clinical Center, Durham VA Medical Center and Duke University, Durham, North Carolina; Neurobiology Research Lab, Durham VA Medical Center and Duke University, Durham, North Carolina; Department of Veterans Affairs, Washington, DC

## Abstract

**Introduction:**

Military service and combat exposure are risk factors for smoking. Although evidence suggests that veterans are interested in tobacco use cessation, little is known about their reasons for quitting, treatment preferences, and perceived barriers to effective tobacco use cessation treatment. Our study objective was to elicit perspectives of Iraq- and Afghanistan-era veterans who had not yet quit smoking postdeployment to inform the development of smoking cessation services for this veteran cohort.

**Methods:**

We conducted 3 focus groups among 20 participants in October 2006 at the Durham Veterans Affairs Medical Center to explore issues on tobacco use and smoking cessation for Iraq- and Afghanistan-era veterans who continued to smoke postdeployment. We used qualitative content analysis to identify major themes and organize data.

**Results:**

Veterans expressed the belief that smoking was a normalized part of military life and described multiple perceived benefits of smoking. Although veterans expressed a high level of interest in quitting, they listed several behavioral, situational, and environmental triggers that derailed smoking cessation. They expressed interest in such cessation treatment features as flexible scheduling, free nicotine replacement therapy, peer support, and family inclusion in treatment.

**Conclusion:**

Our results indicate that the newest cohort of veterans perceives smoking as endemic in military service. However, they want to quit smoking and identified several personal and environmental obstacles that make smoking cessation difficult. Our findings may inform programmatic efforts to increase successful quit attempts in this unique veteran population.

## Introduction

Cigarette smoking is the single greatest cause of illness and death in the United States (1). Military service is a risk factor for smoking (2,3). US military veterans and service members smoke at significantly higher rates than the general population (2,4). Approximately 74% of veterans report a history of cigarette use, compared with 48% in the nonveteran population (3,5). Military service members who experience combat exposure are at even higher risk of initiating or resuming smoking (6). Almost 45% of US service members deployed to Iraq and Afghanistan smoke, which is double the rate of other nonmilitary Americans (7,8).

Most smokers want to quit smoking (9); however, most smokers who try to quit do so without the aid of smoking cessation treatments (9). Only 3% to 5% of smokers who try to quit unaided maintain their quit attempts a year later (10). Increasing the number of successful quit attempts using evidence-based interventions is a public health priority to reduce the number of veterans who smoke.

Although evidence suggests that military personnel and veterans are interested in tobacco use cessation (11,12), little is known about their reasons for quitting, treatment preferences, and perceived barriers to quitting. The objective of our study was to elicit perspectives of Operation Enduring Freedom and Operation Iraqi Freedom (OEF/OIF) veterans who had not yet quit smoking postdeployment to inform the development of smoking cessation services at the Department of Veteran Affairs (VA).

## Methods

We conducted 3 focus groups in October 2006 at the Durham VA Medical Center (VAMC) in Durham, North Carolina. The VAMC institutional review board approved this study.

### Recruitment procedures

To be eligible for the study, participants needed to be current smokers, patients at the Durham VAMC, and OEF/OIF veterans. We identified potential participants through the Durham VAMC electronic medical record system by screening records for military service since September 11, 2001, and current smokers. We then selected a random sample of 199 veterans who met eligibility criteria. We mailed introductory letters that described the study to potential participants, indicating that we might call them to ask for their participation. After the mailing, we further restricted the sample to the 125 veterans living within a 2-hour drive of the Durham VAMC. We attempted to contact by telephone all 125 veterans at least once until 29 eligible veterans agreed to participate. During these recruitment calls, we confirmed eligibility as current smokers and OEF/OIF veterans, further explained study, and scheduled interested veterans for a focus group. Of the veterans we reached by telephone, we excluded 1 because of smoking status, 2 declined to participate, and 4 could not participate because of scheduling conflicts. Of the 29 veterans recruited, 9 did not attend their scheduled focus group; the final sample consisted of 20 veterans.

### Focus group procedures

Each of the 3 focus groups consisted of 6 to 8 participants. Immediately before the focus groups, participants provided informed consent and completed brief surveys on tobacco use and demographics. One member of the study team moderated the groups (K.S.T.), and one member took notes (S.A.). The first focus group included study team observers (J.C.B., P.S.C.). We used a standardized moderator guide consisting of questions on major themes of interest. We audio recorded and transcribed all focus groups. Each group lasted approximately 90 minutes, including time for survey completion. Participants received $50 for focus group participation.

### Measures

The focus group interview guide (Appendix) asked about reasons for using tobacco, solicited views on motivations, barriers, and facilitators to smoking cessation, and garnered ideas to improve smoking cessation services. In addition, we collected information on demographic characteristics (age, race, and sex), former military service status (Active Duty, National Guard, or Reserve), and smoking history, including number of pack-years smoked and level of nicotine dependence. To measure nicotine dependence, we used the 6-item Fagerström Test for Nicotine Dependence (FTND) (13).

### Analysis

We examined focus group information by using qualitative content analysis, which allowed us to code text into "meaning units" that represent important concerns, beliefs, and experiences (14,15). To ascertain meaning units, we first identified emergent codes. Two team members (J.M.G., K.S.T.) independently and manually coded 1 transcript of a focus group discussion, applying 1 or more descriptive codes to chunks of text representing each participant's contributions. The 2 coders compared codes, reconciled differences, and finalized a coding scheme through discussion. A team member (J.M.G.) applied the coding scheme to all of the transcripts, allowing for additional emerging codes, which were refined through discussion with 1 of the original coders (K.S.T.). Through discussion, we organized codes into larger themes and organized data into 4 major topics: reasons for tobacco use during and after deployment, reasons for wanting to quit smoking, perceived barriers to making a quit attempt and maintaining smoking cessation, and facilitators of making successful quit attempts. To illustrate major themes, we selected quotes, identified by focus group (FG1, FG2, FG3). For survey items, we calculated frequencies and means.

## Results

### Sample characteristics

Most participants were male, African American, and veterans of active duty (versus National Guard or Reserves) (Table). Participants reported low nicotine-dependence scores as measured by the FTND, and only 20% reported heavy smoking (≥20 cigarettes/d).

### Qualitative findings

**Why military personnel and veterans use tobacco**

Many veterans expressed the idea that tobacco use was a common and normalized behavior during deployment to Afghanistan and Iraq. "Everyone smoked more when you were over there" (FG2). Three major factors emerged on why military personnel used tobacco during deployment. First, many veterans said they used tobacco during deployment as a way to improve job performance and reduce boredom. They used cigarettes as a way to stay awake during long missions. Smokeless tobacco, however, was used during patrols and at night because tobacco smoke and lit cigarettes could reveal soldiers' locations. Second, military personnel used tobacco as a way to manage stress. Veterans cited tobacco use as a widely accepted justification for taking breaks. "The only way I can pause . . . is if I go take a smoke break" (FG2). Also, smoking offered soldiers a way to escape from their situation to "take your mind off the horrible place you're at" (FG2). Lastly, veterans said they used tobacco during deployment as a way to foster social connections. "There is a lot of camaraderie around smoking" (FG2). Designated smoking areas were a popular place to share information. "I smoked primarily as a way to maintain communication. The best way to get information and disseminate it was smoking areas" (FG1).Veterans used smoking as a reason to gather and offer silent support after the death of a fellow soldier. "We'd know one of the guys didn't come back and We'd all sit there and smoke and nobody would say a word" (FG1).

Once soldiers returned stateside, they continued to smoke as a way to modulate negative moods (eg, anger dysregulation, irritability, stress). "If I have that stress in my life I'm gonna go spend that money to have that cigarette that's gonna help calm me down before I go off on somebody for no reason" (FG3). Many veterans also cited difficulty coping with a postdeployment shift to civilian life as a reason for continuing to smoke. "Smoking is a comfort" (FG1). Veterans cited combat-related injuries, unstructured life outside of the military, sleep disorders, and inability to turn off the military mindset (eg, hypervigilance) as reasons for civilian-transition difficulties that triggered smoking. Lastly, like other nonveteran groups, participants cited tobacco addiction as a reason for continuing to smoke.

**Why veterans want to quit smoking**

Most veterans expressed desires to stop using tobacco; 5 major themes emerged. First, participants cited personal health as a major reason for wanting to quit using tobacco. As a young participant stated, "Well, I want to be able to breathe. I'm not trying to be funny, like, I want to be able to actually do the physical things I used to be able to do and not get all out of breath and red in the face cause that's kind of embarrassing to be as young as I am and I used to be in the military and I'm all huffing and puffing for breath" (FG3). Improved personal health also extended to long-term health concerns of illnesses, such as cancer, as a major motivator to quit smoking.

Veterans cited becoming weary of being dependent upon cigarettes as a motivator for smoking cessation. "I'm tired of being chained to it [smoking]" (FG3). Another participant stated, "I think it is a disgusting habit. I don't like waking up in the morning and feeling like I got to have a cigarette in my mouth" (FG2). Side effects of smoking, such as staining of teeth and hands, bad breath, and making one's home dirty from cigarettes, strengthened veterans' personal determination to quit.

Family also served as a reason for many young veterans to become committed nonsmokers. Some participants did not want their children to see them smoke or wanted to avoid their children's exposure to secondhand smoke. Others cited personal experiences of seeing loved ones die of smoking-related illnesses and wanting to protect their loved ones from similar trauma. "My family is my biggest reason to quit. . . . I watched my grandfather die of lung cancer last year literally until he took his final breath and I will not let my children see me die that way" (FG1).

Veterans cited the cost of cigarettes and shifting social norms on smoking as strong environmental cues to become nonsmokers. "It's getting too expensive to smoke" (FG3). Social pressure to become a nonsmoker seemed to extend to all areas of veterans' lives stateside. "The rest of the entire world has somehow revolved around this entire antismoking ban" (FG1).

**Why it is difficult for veterans to become nonsmokers**

Veterans listed several situational, behavioral, and environmental triggers that made it difficult to maintain quit attempts. Some participants said they were unaccustomed to their unstructured daily lives after structured military life and, therefore, smoked to fill the time. Others said it was difficult to break the habit because smoking was linked to so many of their other life activities, such as driving, eating, and drinking alcohol. Being around friends and family who used tobacco was a commonly cited barrier to smoking cessation. Veterans also said feelings of depression, irritability, uncontrolled anger, and sleeplessness made smoking cessation difficult. Some participants said they experienced side effects from using nicotine replacement therapy (NRT) and other cessation pharmacotherapies, which prompted smoking relapse. Lastly, many veterans said another deployment derailed a quit attempt.

**What would facilitate veteran efforts to quit smoking**

Focus group participants offered several recommendations for improving programs to help them make a quit attempt and maintain smoking cessation. Overall, they expressed a need for a personalized approach for smoking cessation services. "A one-size-fits-all I don't think is going to work for smoking at all" (FG2). Specifically, veterans wanted free or reduced-cost NRT and other smoking cessation pharmacotherapies and suggested offering innovative incentives to quit smoking, such as gas, grocery coupons, or cash. Participants also said they required smoking cessation services that were convenient and accessible; they cited frustrations with smoking cessation classes offered only during regular working hours and with long waits for class enrollment. They expressed an interest in smoking cessation telephone counseling but found quitlines to be impersonal. As an alternative, they suggested personalized telephone counseling, with the option to supplement calls with in-person counseling sessions. Some veterans expressed interest in a smoking cessation peer-support program that pairs them with successful veteran ex-smokers. Lastly, participants expressed interest in providing family or household members with access to treatment.

## Discussion

Tobacco use has been a part of military culture since World War I, when cigarettes became widely available; service members were issued cigarettes with their rations to help them escape the tedium of war, boost morale, and offer pleasure, comfort, and currency (16). Our results show that smoking is still perceived as endemic in military service by the newest cohort of veterans. Moreover, we found that OEF/OIF veterans felt smoking was an encouraged and normalized part of life during deployment. Our results are consistent with previous findings among active-duty service members. Deployed troops have higher rates of smoking initiation and smoking relapse compared with nondeployed troops (6).

Prior research shows that smoking is a way to manage stress, boredom, anxiety, and sleep deprivation among active-duty military personnel (17,18). Our results extend this research. Veterans described additional perceived benefits of smoking during their deployment, including creating a sense of camaraderie, facilitating communication outside one's work area, being able to take approved work breaks to smoke, and improving job performance. Instead of smoking, military service members should be offered access to healthy activities that foster a sense of troop cohesion while alleviating stress and boredom. To counter perceptions that tobacco use improves job performance, efforts should be made to increase soldiers' awareness of the association between smoking and risk of injury during physical training (19,20) and reinforce their beliefs that smokers present a risk to other service members during deployment because of reduced levels of readiness caused by withdrawal symptoms and lit cigarettes revealing locations (18).

Our findings suggest that veterans continue to use tobacco to modulate depressed mood, anxiety, and boredom after returning home. Feelings of stress related to interpersonal relationships (eg, family, community) are also prevalent among returning combat veterans (21,22). Smokers in our study reported using cigarette breaks as a way to deal with anger, by stepping away from escalating situations with others. When asked why quitting smoking was so difficult for them, many veterans listed symptoms consistent with depressive disorders and posttraumatic stress disorder (PTSD) (eg, irritability, uncontrolled anger, sleeplessness). Our findings align with other research; 37% of all OEF/OIF veterans seen in VA health care facilities received mental health diagnoses (23). People with mental health issues are more likely to smoke and may experience more difficulty when trying to quit (24,25). For example, people with PTSD are more likely to be smokers and smoke more heavily than smokers without PTSD (26). The VA successfully integrated tobacco use cessation treatment into PTSD mental health services (27). Further efforts should be made to integrate smoking cessation treatments into other health care services accessed by veterans.

Despite the multiple challenges OEF/OIF veterans expressed, our results indicate that these veterans have a strong desire to quit using tobacco. This finding is consistent with other research; almost 70% of veteran smokers want to quit (12). Since 2002, the VA health care system has implemented an array of systemwide evidence-based policies and programs to facilitate smoking cessation efforts (4). These included such changes as increased access to smoking cessation pharmacotherapies and elimination of copayments for outpatient smoking cessation counseling; these positive changes contributed to an increase of approximately 60% in NRT and buproprion prescriptions from 2004 to 2008 (28). Moreover, virtually all VAs offer some form of a tobacco control program, and most veterans seen in the VA for care are screened for tobacco use and provided with brief cessation counseling (11,29). Although empirically based smoking cessations services are available free at the VA, many of the participants in our study reported not knowing services were available, suggesting an opportunity to improve marketing of existing VA smoking cessation services.

Our findings should be interpreted with caution. A regional cohort limits the generalizability of our findings; the results may not represent the needs and preferences of veterans living outside the southeastern US region or veterans not seeking VA care. Furthermore, OEF/OIF veterans may have unique smoking needs and preferences that may not translate to other veteran cohorts. Also, we were not able to directly assess psychiatric diagnoses in this cohort. Future studies should include full mental health history and include more geographically diverse samples.

Smoking is prevalent in military service and is a behavior that carries over into civilian life. We found that OEF/OIF veterans want to quit smoking but have multiple behavioral, situational, and environmental triggers that make smoking cessation complex. In addition, these veterans are younger overall than past cohorts of veterans seeking VA care (23,30). Thus, these veterans often have young families and are engaged in school and work. Future smoking cessation strategies for OEF/OIF veterans may need to promote themes that have not been used for previous cohorts (eg, quit for the sake of children, increase physical stamina). This younger cohort may also be more likely to use new technologies to get help. The Department of Defense website, Quit Tobacco — Make Everyone Proud (www.ucanquit2.org), provides online assistance with live chat services and individualized quit plans. The Department of Defense and the VA have partnered to extend access for this online resource to veterans enrolled for care in the VA to target the smoking cessation needs of OEF/OIF veterans. Themes from our analysis may help serve as a foundation to reach, engage, and facilitate successful quit attempts in this unique veteran population.

## Acknowledgments

This work was supported by a grant for the VA Office of Public Health and Prevention. At the time of this analysis, Dr Gierisch was funded by an Agency for Healthcare Research and Quality National Research Service Award at Duke University Medical Center (grant no. T-32-HS000079).

## Figures and Tables

**Table.** Characteristics of Iraq- and Afghanistan-Era Veterans (N = 20) Participating in Focus Groups on Tobacco Use, Durham, North Carolina, 2006

**Characteristic**	Value **a**
**Male, n**	17
**Age, mean (SD), y**	34.8 (9.5)
**Race, n**	
White	7
African American	11
Native American	1
Not reported by participant	1
**Former military service status, n**	
Reserve	1
National Guard	4
Active Duty	13
Not reported by participant	2
**Packs-years,^b^ mean (SD), n**	11.9 (14.3)
**Fagerström Test for Nicotine Dependence,^c^ mean score (SD)**	4.3 (2.2)

Abbreviation: SD, standard deviation.

a Mean values exclude participants with missing data (age, 1; military service status, 2; pack-years, 2).

b One pack-year is the equivalent of smoking 20 cigarettes per day for 1 year.

c Test score options ranged from 0 to 10; high level of dependence was defined as a score ≥6.

## Appendix. Veteran Focus Group Moderator Guide: Tobacco Use and Cessation Among Returning Veterans (N = 20) of Operation Enduring Freedom and Operation Iraqi Freedom, Durham Veterans Affairs Medical Center, North Carolina, 2006

**Introduction**

Hello, everyone. Thank you for taking time out of your busy schedules to talk to us today.

I am ______. We have been working with veterans to find out about their use of cigarettes and other tobacco products and their experiences with trying to quit. Also, in the room is_____ who will be writing things on the flipcharts and _____ who will be taking notes about what seem to be the most important issues that we discuss. All of us will keep the discussions confidential.

On behalf of myself and the staff at the VA, I want to express our appreciation for your service to the country. Thank you.

**Purpose**

Our primary purpose today is to discuss your experiences with tobacco both during deployment and after you came home. Your issues, comments, and recommendations are very important to us and we are here to learn from you. Therefore, I am going to do as little talking as possible.

I will be asking some questions, asking for more information on certain topics, and generally moderating the discussion. There are no right or wrong answers — it's your opinions and thoughts that are important to us.

**Procedure**

Before we get started, I would like to talk about the process.

- First, everything we talk about is confidential.
- Second, your participation is voluntary. If you don't want to participate in part of the discussion, you don't have to.
- Third, we will audiotape the discussion to make sure we get all the information you provide.
- Fourth, I am interested in hearing from everyone here. So, at times I may call on you directly to get your opinion. At other times, I may need to interrupt so that I can hear from others or to move us along to the next question.
- Because this will be audiotaped, this works best if only one person speaks at a time.

Are there any questions before we get started?

1. (Ice Breaker). First, I'd like to hear *briefly* about your deployment experiences. Can you tell me your branch and component of service (eg, Army or Marines, Reserve, National Guard, or Active Duty) and how long were you deployed?
  Probe: When did you last return from deployment?
2. Did you smoke in the military or use chewing tobacco? Please tell us a little about this including when and why you smoked or used chewing tobacco.
  Probe: Are you still smoking?
  Probe: How has your smoking changed, if at all, since you've been home?
3a. What reasons might be important enough to you to quit smoking?
  Probe: Which of the concerns we've just talked about are MOST important to you? (UNDERLINE or * on the flipchart)
3b. What messages might influence you to consider quitting? These could be communications from your doctors, friends/family, peers, media?
  Probe: Which of the messages we've just talked about are MOST important to you? (UNDERLINE or * on the flipchart)
3c. What do you think would help you make a quit attempt?
  Probe: What would help you make use of VA services for smoking cessation?
  Probe: What has helped you make quit attempts in the past?
3d. What would make smoking cessation treatment more attractive?
  Probe: What would be included in a program that would be attractive?
4. What got in the way of previous attempts to quit?
  Probe: What barriers have you encountered when trying to quit?
5. What could the VA do to help you or someone you know stop smoking or using tobacco products?

That's all the time we have. Again, I would like to thank you for your time tonight and for your service to our country.
